# Bioactive Flavonoids and Catechols as Hif1 and Nrf2 Protein Stabilizers - Implications for Parkinson’s Disease

**DOI:** 10.14336/AD.2016.0505

**Published:** 2016-12-01

**Authors:** Natalya A. Smirnova, Navneet Ammal Kaidery, Dmitry M. Hushpulian, Ilay I. Rakhman, Andrey A. Poloznikov, Vladimir I. Tishkov, Saravanan S. Karuppagounder, Irina N. Gaisina, Anton Pekcec, Klaus Van Leyen, Sergey V. Kazakov, Lichuan Yang, Bobby Thomas, Rajiv R. Ratan, Irina G. Gazaryan

**Affiliations:** ^1^Burke Medical Research Institute, Weill Medical College of Cornell University, White Plains, NY 10605, USA; ^2^D. Rogachev Federal Scientific and Clinical Center for Pediatric Hematology, Oncology, and Immunology, Moscow 117997, Russia; ^3^Departments of Pharmacology, Toxicology & Neurology, Medical College of Georgia, Augusta University, Augusta, GA 30912, USA; ^4^ValentaPharm, Moscow 119530, Russia; ^5^Department of Chemical Enzymology, Moscow State University, Moscow 119992, Russia; ^6^Department of Medicinal Chemistry and Pharmacology, University of Illinois at Chicago, Chicago, IL 60612, USA; ^7^Neuroprotection Research Laboratory, Department of Radiology, Massachusetts General Hospital, Harvard Medical School, Charlestown, MA 02129, USA; ^8^Department of Chemistry and Physical Sciences, Dyson College, Pace University, Pleasantville, NY 10570, USA.

**Keywords:** Parkinson’s disease model, glutathione depletion model, HIF prolyl hydroxylase, lipoxygenase, fisetin, luteolin, Keap1

## Abstract

Flavonoids are known to trigger the intrinsic genetic adaptive programs to hypoxic or oxidative stress via estrogen receptor engagement or upstream kinase activation. To reveal specific structural requirements for direct stabilization of the transcription factors responsible for triggering the antihypoxic and antioxidant programs, we studied flavones, isoflavones and catechols including dihydroxybenzoate, didox, levodopa, and nordihydroguaiaretic acid (NDGA), using novel luciferase-based reporters specific for the first step in HIF1 or Nrf2 protein stabilization. Distinct structural requirements for either transcription factor stabilization have been found: as expected, these requirements for activation of HIF ODD-luc reporter correlate with *in silico* binding to HIF prolyl hydroxylase. By contrast, stabilization of Nrf2 requires the presence of 3,4-dihydroxy- (catechol) groups. Thus, only some but not all flavonoids are direct activators of the hypoxic and antioxidant genetic programs. NDGA from the Creosote bush resembles the best flavonoids in their ability to directly stabilize HIF1 and Nrf2 and is superior with respect to LOX inhibition thus favoring this compound over others. Given much higher bioavailability and stability of NDGA than any flavonoid, NDGA has been tested in a 1-methyl-4-phenyl-1,2,3,6-tetrahydropyridine (MPTP)-animal model of Parkinson’s Disease and demonstrated neuroprotective effects.

Flavonoids are one of the largest families of natural products. It is supposed that the consumption of flavonoid-rich foods throughout life holds the potential to lower the risks of cancer, heart attack, and stroke, as well as limit age-related neurodegeneration [1]. Classically, the biological actions of flavonoids have been attributed to their antioxidant properties and kinase signaling cascades [2, 3]. However, they can barely compete with non-specific antioxidants such as ascorbic acid and α-tocopherol, which are present at 100-fold higher concentrations in plasma and organs than any flavonoid. Recent studies clearly point to the structure-activity relationships (SAR) for protein kinase inhibition by flavonoids [4], and distinct structural requirements for binding and activation of α- and β-estrogen receptors [5]. Some flavonoids have the so called catechol (3’,4’-dihydroxy) motif on the freely rotating phenyl ring (shown in red in Table 1). Flavonoids are also known to activate anti-hypoxic and anti-oxidant genetic programs in the cell, and in particular those triggered by HIF (Hypoxia Inducible Factor) and Nrf2 transcription factors.

**HIF** is a widespread transcription factor activating a battery of genes involved in glucose uptake and metabolism, extracellular pH control, angiogenesis, erythropoiesis, mitogenesis, and apoptosis. HIF1 consists of 2 subunits, among which HIF1-α is rapidly degraded under normoxic conditions: HIF1-α levels are regulated by post-translational modification such as phosphorylation, acetylation, and hydroxylation. The latter is considered as the major regulator of HIF1-α protein stability: hydroxylation of Pro564 and/or 402 residues in HIF-1α is a prerequisite for the interaction with the tumor suppressor von Hippel-Lindau (VHL) protein yielding a complex that provides HIF ubiquitination and subsequent proteasomal degradation (see review [6] and ref therein). HIF hydroxylation is executed by α-ketoglutarate (αKG) dependent non-heme iron dioxygenases, the so-called prolyl hydroxylases (HIF PHD1-3 isozymes). Their specific inhibitors are currently in the focus of drug discovery efforts pursued by many companies (Fibrogen, Amgen, J & J, and P & G). Recently, two groups independently provided direct evidence for quercetin being a HIF PHD2 inhibitor with an apparent inhibition constant of 10 µM [7, 8], and baicalein working as a PHD2 inhibitor (Ki = 7 µM) competitive with α-KG [9]. However, *in vitro* enzyme assay does not answer clearly which flavonoids in particular are best inhibitors of HIF PHDs.

**12/15-LOX** (12/15-lipoxygenase), like HIF PHDs, is a non-heme iron dioxygenase, however, it oxidizes lipids (not transcription factors) and thus, has a much narrower access to the active site as compared to HIF PHDs. Baicalein [10, 11] and NDGA [12] are classical but fairly non-selective LOX inhibitors, as well as several other flavonoids [13]. LOX, originally isolated as a factor that mediates mitochondrial breakdown in the development of red blood cells [14], is now known to contribute to neuronal cell death *in vitro* [15, 16] and *in vivo* [17, 18]. In recent years, 12/15-LOX inhibitors are being tested in animal models of stroke where they consistently reduce ischemic injury, blocking neuronal cell death and edema formation [19]. Therefore, it was interesting to compare the inhibitory properties of flavonoids and NDGA with respect to both enzymes, HIF PHD and 12/15-LOX.

**Nrf2** (nuclear factor erythroid 2-related factor2) is a key transcription factor orchestrating the antioxidant program by inducing the expression of pro-survival proteins and cytoprotective enzymes such as thioredoxin reductase, glutathione reductase, glutathione *S*-transferase (GST), hemeoxygenase-1 (HO1), catalase, etc. Nrf2 is sequestered under homeostatic conditions by binding to its inhibitory protein, Keap1 (Kelch-like ECH-associated protein-1) [20, 21]. Keap1 serves as a bridge between Nrf2 and the Cul3-Rbx1 E3 ubiquitin ligase, leading to polyubiquitination of the lysines positioned within the central α-helix of the Neh2 domain under homeostatic conditions [22-24]. Upon oxidative/electrophilic stress, Keap 1 undergoes modification of some of its active cysteine residues. As a result of Keap 1 conformational changes, Nrf2 protein is released from its complex with Keap1 and translocates to the nucleus, where it forms heterodimers with other transcription regulators, and induces the expression of antioxidant genes controlled by promoters with the antioxidant response element (ARE) [25]. Many flavonoids are known to activate Nrf2 although the detailed mechanism of their action is unknown. There is some evidence for two modes of Nrf2 activation by flavonoids: (a) direct, by forming a semiquinone radical which, in turn, activate Nrf2 by modifying Keap1 thiols, and (b) indirect, via activation of various kinase pathways resulting in phosphorylation of Nrf2 and subsequent induction of Nrf2-dependent genes.

A novel approach to high throughput screening (HTS) for stabilizers of transcription factors developed in our laboratory is based on stable expression of a fusion between luciferase and a transcription factor minimum domain (Fig. 1). Minimum domain is a portion of a transcription factor that is necessary and sufficient for recognition, modification and subsequent ubiquitinylation to occur. Under steady-state conditions, the background luminescent signal equals to the sum of all forms of luc-labeled surrogate of a transcription factor except for its proteolytic fragments: [MD-luc]= K_o_Σ (1/k_i_) (Fig. 1). HIF prolyl hydroxylase (PHD) [26] controls the rate-limiting step in HIF1-ODD luc reporter activation, whereas Neh2-Keap1 interaction [27] controls such step in Neh2-luc reporter activation. Hence, either reporter permits selection of direct stabilizers of the corresponding transcription factor. The newly developed assays [26] and [27], for the first time provide an opportunity to monitor direct effects of flavonoids on stability of HIF1 or Nrf2 transcription factors, respectively, in the form of their luciferase fusion. As we show below, there are distinct structural requirements for stabilization of either transcription factor by flavonoids and biologically active catechols, meaning that not all but only specified compounds are direct triggers of survival programs.

**Figure 1.**
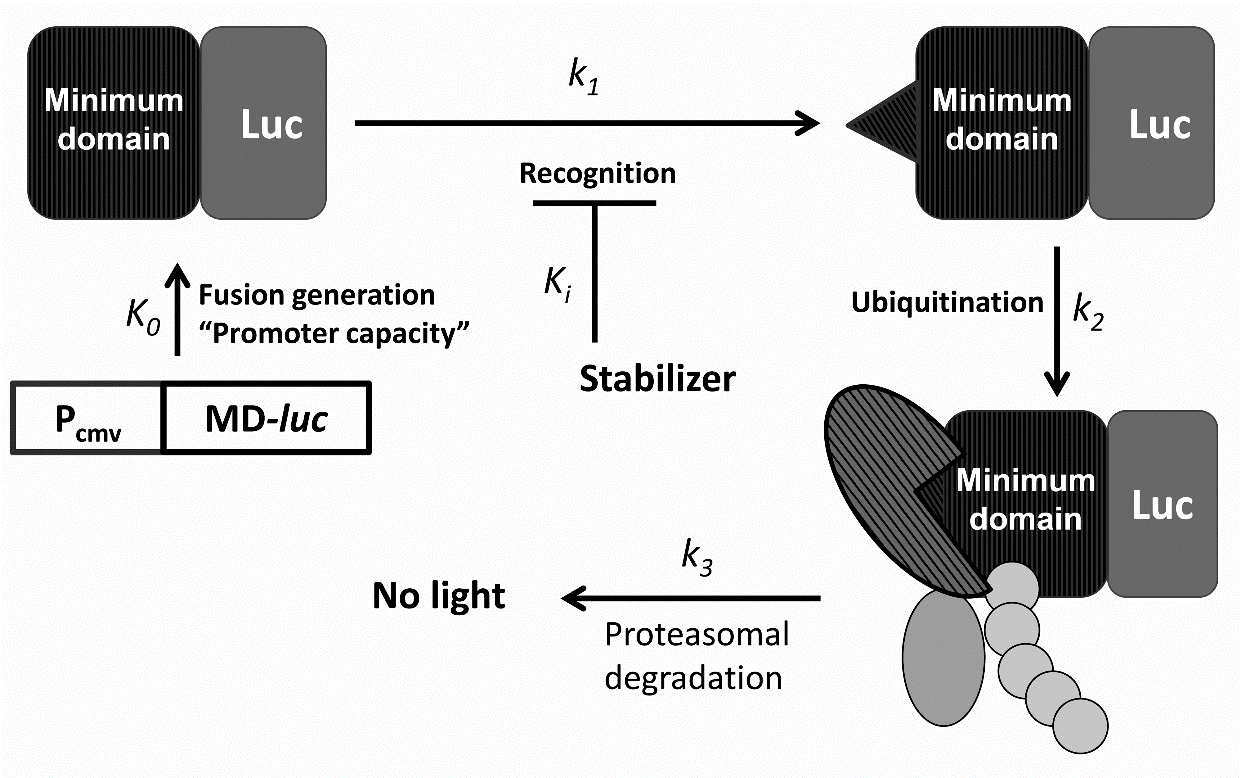
**Schematic presentation of a luciferase-labeled surrogate transcription factor reporter**. K_o_, rate of fusion protein generation, “promoter capacity”, k_1_, rate constant for the recognition step, which has been shown to be rate-limiting for the reporters under discussion, K_i_, inhibition constant for a stabilizer working at the first step; k_2_, ubiquitinylation rate constant; k_3_, proteasomal degradation rate constant.

## MATERIALS AND METHODS

### Reagents

Flavonoids were purchased from Indofine Chem Co., Inc. (Hillsborough, NJ), FG-4592 was from Selleckchem (Houston, TX), Didox from Cayman Chemical Co. (Ann Arbor, MI). All other reagents used were from Sigma (St. Louis, MO).

### Determination of iron binding constant

Calcein displacement assay in the presence of varied concentrations of flavonoids and catechols has been used. Calcein release from its complex with iron was monitored by fluorescence as described in [26].

### Determination of rate constant for non-enzymatic ferricyanide reduction

The rate of ferricyanide reduction by individual flavonoids was measured spectrophotometrically at 420 nm with the fixed concentration of ferricyanide (1 mM) and varied concentrations of a flavonoid in Potassium-phosphate buffer, pH 7.0. The rate constant for direct ferricyanide reduction was calculated from the slope of the dependence reaction rate vs flavonoid concentration and used as a measure of flavonoid non-specific reducing power.

### Rabbit LOX 12/15 (Rabbit Reticulocyte 15-LOX-1) activity

The activity of the purified enzyme was measured in the end-point *in vitro* assay of arachidonic acid oxidation following the formation of fluorescent diphenyl-1-pyrenylphosphine oxide [28]. DMSO was used as a control. The compounds were tested at 25 µM concentration.

### HIF1 ODD-luc and Neh2-luc reporter assays

SH-SY5Y cell lines stably expressing HIF1 ODD-luc [26] or Neh2-luc [27] reporters were grown in the DMEM/F12 supplemented with GlutaMAX (Thermo Fisher Scientific) containing 10% FBS, 100 U/ml penicillin, and 100 μg/ml streptomycin and plated into 384-well white flat-bottom plates at 7,000 cell/well in 30 µl serum and incubated overnight at 37°C, 5% CO_2_. The next day compounds were added to a final concentration of 10 µM and the plates were incubated for 3 hours at 37°C. Reporter activation after 3 h incubation with the studied compound was compared to that for ciclopirox (HIF1 ODD-luc reporter) or TBHQ (Neh2-luc reporter), taken as 100%. Luciferase reporter activity was measured with a SteadyGlo reagent from Promega providing stable reading for 1 h.

### Extended SAR Analysis

Selected hits were tested in 96-format white, flat-bottom plates with varied concentrations of an inhibitor (0.5-25 µM). Cells were plated at the density of 25,000 cell per well using a WellMate multichannel dispenser from Matrix (Thermo Fisher Scientific, Waltham, MA) and grown overnight on DMEM/F12+GlutaMAX (100 µl per well). Then an inhibitor was added, and the plates were incubated for a fixed time interval; the medium was removed, cells lysed, and luciferase activity was measured on a SpectraMax M5e platereader (Molecular Devices, Sunnyvale, CA) with BrightGlo™ reagent (Promega, Madison, WI). The reporter activation was normalized to the background luminescence. The effect of 0.5 mM N-acetylcysteine (NAC) was studied by simultaneous addition of 5 µL of 10 mM stock of NAC in water and 2 µL of 50x stock solutions of a flavonoid in DMSO.

### HRE-luc reporter assay

A promoter-reporter construct that contained 68 bp of a known hypoxia and HIF-1 regulated gene, enolase, containing a wild type hypoxia response element (HRE, 5’-RCTGT-3’), is a widely used approach for screening for HIF activators of different mechanism of action [29]. A cell-based assay with HRE-luciferase reporter system is based on transfected immortalized hippocampal cell line (HT22) and allows screening for a broad spectrum of compounds that include: activators of HIF transcription; activators of HIF binding to HRE; and effectors of HIF protein stability (PHD inhibitors, pVHL & proteasome inhibitors). The assay was performed the same way as for HIF ODD-luc reporter except the cells were plated at the density of 10,000 cell per well and incubated overnight.

### Glutathione depletion model and Viability Assay

Primary neuronal cultures were prepared from the forebrains of Sprague-Dawley rat embryos (E17) and plated on 96 well plates at a 10^6^ cells/ml density. After 24 hours, cells were rinsed with warm PBS and then placed in minimum Essential Medium (MEM; Life Technologies, Grand Island, NY) containing 5 mM HCA in the presence of a compound of interest at varied concentrations. Cells were incubated for 24 h or longer to see 90% cell death in HCA treated controls. Viability was assessed by the MTT (4,5-dimethylthiazol-2-yl)-2,5-diphenyltetrazolium bromide) assay.

### RNA isolation and real-time RT-PCR

For VEGF gene expression studies, total RNA was isolated from immature primary cortical neurons, pretreated overnight with the indicated concentration of a bioactive compound, using the NucleoSpin RNA II kit (Macherey-Nagel, Bethlehem, PA) according to manufacturer’s protocol. Real-time PCR was performed in triplicate as a duplex reaction using a VEGF (Mm01281449_m1) gene expression assay with a 6-carboxyfluorescein-labeled probe, and a β actin gene expression assay with a VIC-labeled probe (Applied Biosystems, Foster City, CA), so that gene amplification could be normalized to β actin. These experiments were performed using a 7500 Real-time PCR System (Applied Biosystems) using standard PCR protocol and amplification conditions.

### Animals

Mice were housed and treated in strict accordance with the NIH *Guide for the Care and Use of Laboratory Animals*. The Institutional Animal Care and Use Committees of the Weill Medical College of Cornell University, New York and Medical College of Georgia, Augusta University, Augusta approved all procedures. Mice were maintained in a pathogen-free facility and exposed to a 12 h light/dark cycle with food and water provided *ad libitum*. C57Bl6 mice were procured from Jackson laboratories (Bar Harbor, ME).

### MPTP and NDGA administration in mice for neuroprotective studies

Acute MPTP-intoxication paradigm was used to test the neuroprotective effects of NDGA using male C57Bl6 mice. In this protocol, 10-week-old C57Bl6 mice (*n* = 8-10 per group) were divided into four different groups consisting of 1) a control group treated with saline alone; 2) a group treated with MPTP alone; 3) a group treated with NDGA alone and; 4) group treated with NDGA in combination with MPTP. NDGA was dissolved in 1:4 ethanol:neobee oil (a derivative of coconut oil). MPTP 10 mg/kg free base was administered intraperitoneally three times a day every two hours. Animals with NDGA in combination with MPTP were administered NDGA by oral gavage with 50 and 100mg/kg/day body weight of the drug in 100 microliter volume once a day for 4 days before MPTP and once a day for 3 days after MPTP. On the day of MPTP NDGA was administered to mice 2 hours before the initiation of MPTP. Animals belonging to MPTP and control groups received the vehicle (ethanol:neobee oil, 1:4) whereas the drug alone group received respective doses of NDGA at the same frequency. Animals belonging to MPTP and control groups received the vehicle (ethanol:neobee oil, 1:4) whereas the drug alone group received respective doses of NDGA at the same frequency. All animals were sacrificed on the 7^th^ day after MPTP.

### Measurement of striatal levels of catecholamines and MPP+ by HPLC

Striatal levels of dopamine (DA) and its metabolites 3, 4-dihydroxyphenylacetic acid (DOPAC), and homovanillic acid (HVA) were measured after sonication and centrifugation in chilled 0.1 M perchloric acid (PCA, 100 µl/mg tissue) as previously described. Briefly, 15 µl supernatant was isocratically eluted through an 80 x 4.6 mm C18 column (ESA, Inc. Chelmsford, MA) with a mobile phase containing 0.1 M LiH_2_PO_4_, 0.85 mM 1-octanesulfonic acid and 10% (v/v) methanol and detected by a 2-channel Coulochem II electrochemical detector (ESA, Inc. Chelmsford, MA). Concentrations of dopamine, DOPAC and HVA are expressed as ng per mg protein. The protein concentrations of tissue homogenates were measured according to BCA assay (Pierce Biotech). For MPP+ measurement NDGA was administered at a dose of 100mg/kg/day once daily for four days followed by a dose on the 5^th^ day 30 minutes before MPTP (30mg/kg free base). Striatal tissues were sonicated and centrifuged in 0.1 M PCA and an aliquot of supernatant was injected onto a Brownlee aquapore x 03-224 cation exchange column (Rainin, Woburn, MA). Samples were eluted isocratically with 20 mM boric acid-sodium borate buffer, pH 7.75, containing 3 mM tetrabutylammonium hydrogen sulfate, 0.25 mM 1-heptanesulfonic acid and 10% isopropanol. MPP^+^ levels were detected with a fluorescence detector set by excitation at 295 nm and emission at 375nm [30].

### Immunohistochemistry and morphometric analysis

Mice were anesthetized with sodium pentobarbital, transcardially perfused with 0.9% saline followed by 4% paraformaldehyde in 0.1 M PBS, pH 7.4. Brains were dissected out, post fixed in 4% paraformaldehyde for 24 h and cryopreserved in 30% sucrose/PBS for 48 h. Snap-frozen brains were coronally sectioned at 40 µm thickness encompassing the substantia nigra using a cryostat. Briefly, sections were rinsed in PBS and incubated in 3% hydrogen peroxide/10% methanol solution for 10 min to quench endogenous peroxidase activity. Sections were permeabilized/blocked in 10% normal goat serum (NGS)/0.1% Triton X-100/PBS for 1 h at room temperature. Sections were incubated overnight at 4°C with the following primary antibodies in PBS containing 2% NGS/0.01% Triton X-100: rabbit polyclonal anti-tyrosine hydroxylase (TH) (1:1000) (Novus Biologicals, Littleton, CO). Biotinylated secondary antibodies (Jackson ImmunoResearch Laboratories Inc.) were used appropriately after incubation with streptavidin ABC solution (Vector Laboratories, Burlingame, CA). Immunostaining was visualized by diaminobenzidine (Sigma, St Louis, MO) chromogen. Sections were mounted on glass, dehydrated and cover slipped with cytoseal (Thermo Scientific, Waltham, MA). Digital images were captured with Coolpix 5000 Nikon Camera. Tyrosine hydroxylase immunostained sections were counterstained with thionin before dehydration and cover slipped with cytoseal. Nissl (thionin)-stained and tyrosine hydroxylase-positive neuronal counts were estimated within the substantia nigra by Stereoinvestigator software (Microbrightfield) as previously described [30].

### Computer modeling

Docking experiments were performed using the CDOCKER algorithm as implemented in the Discovery studio 2.5 software suite (Accelrys, San Diego, CA), followed by force field minimization and binding energy calculations using the PHD2 crystal structure with the bound inhibitor (2G19.pdb) as the starting template structure. Preparation of the receptor was done by running a protein check and identifying all the elements of the structure. It was noted that there were amino acids missing on the N-terminus and C-terminus, however these were not in close proximity to the binding site and therefore there was no need to add them to the structure. Force field minimization was carried out using the molecular mechanics algorithm CHARMm as implemented in Discovery Studio 2.5.

### Statistical Analysis

All *in vitro* assays were performed at least in triplicate, and presented in Table as mean ± SD. Results of *in vivo* experiments were expressed as means ± SEM or mean ± STDEV. Significance was determined by one-way or two-way ANOVA followed by the Student-Newman-Keuls test or a two-tailed unpaired Student *t* test. Significance was set at *P* ≤ 0.05. All statistical analyses were performed using the Prism software (GraphPad, San Diego, CA).

**Table 1A:** Comparison of flavones performance in reporter activation assays. Original data in the table represent Mean ± SD. Catechol (3’,4’-dihydroxy) motif on the freely rotating phenyl ring is shown in red.

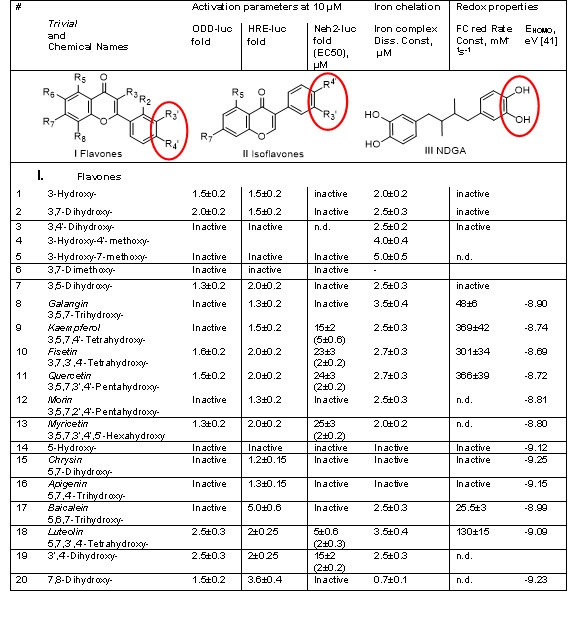

**Table 1B:** Comparison of isoflavones and catechols performance in reporter activation assays. Original data in the table represent Mean ± SD.

#	*Trivial* and Chemical Names	Activation parameters at 10 µM			Iron chelation	Redox properties	
		ODD-luc fold	HRE-luc fold	Neh2-luc fold (EC50), µM	Iron complex Diss. Const, µM	FC red Rate Const, mM^-1^s^-1^	E_HOMO_, eV [41]
**II. Isoflavones**							
21	*Methoxyvone* 5-Methyl-7-methoxy-	Inactive	3.2±0.35	Inactive	Inactive	n.d.	
22	*Ipriflavone* 7-Isopropoxy-3-phenyl-	Inactive	2.5±0.3	Inactive	Inactive	n.d.	
23	*Genistein* 6,7,4’-Trihydroxy-	Inactive	1.5±0.2	Inactive	Inactive	n.d.	
24	*5-Hydroxy-daidzein* 5,7,4’-Trihydroxy-	Inactive	Inactive	Inactive	Inactive	n.d.	
25	*8-Hydroxy-daidzein* 7,8,4’-Trihydroxy-	Inactive	Inactive	Inactive	1.5±0.3	n.d.	
26	*Daidzein* 7,4’-Dihydroxy-	Inactive	Inactive	Inactive	Inactive	n.d.	
27	*3’-Hydroxy-daidzein* 7,3’,4’-Trihydroxy-	2.5±0.3	2.5±0.3	15±2 5±0.6	3.5±0.4	n.d.	
**III. Catechols**							
28	*NDGA* 4,4'-(2,3-dimethylbutane-1,4-diyl)dibenzene-1,2-diol	2.5±0.3	2±0.2	15±1.8 (2±0.3)	2.5±0.3	n.d.	
29	*Levodopa* 3,4-Dihydroxy-L-phenylalanine	2.0±0.25	1.7±0.2	Inactive (20±3)	2.5±0.3	n.d.	
30	*D-DOPA* 3,4-Dihydroxy-D-phenylalanine	Inactive	Inactive	Inactive (20±3)	2.5±0.3	n.d.	
31	*Carbidopa* N-Aminomethyldopa	Inactive	Inactive	Inactive (20±3)	2.5±0.3	n.d.	
32	*DHB* Ethyl 3,4-dihydroxybenzoate	Active above 50 µM	Active above 20 µM	Inactive (20±3)	2.5±0.3	n.d.	
33	*Didox* 3,4-Dihydroxy-benzohydroxamate	Active above 100 µM	Active above 100 µM	Inactive (100±5)	1.5±0.3	n.d.	
**IV. Other**							
34	*Calcein*	2.0±0.25	1.7±0.2	Inactive	0.05±0.01	n.d.	

### RESULTS

The Spectrum library of FDA approved drugs and biologically active compounds contains a large variety of flavonoids (80 flavones, 90 isoflavones, 16 flavanones). To derive structure-activity relationship (SAR), activation parameters for flavones and isoflavones of interest (purchased in the form of powder) were determined from titration curves for all reporter assays (Table 1A&B). In the case of HIF1 ODD-luc and Neh2-luc reporter assays, only a limited number of compounds showed activation at 3 hr incubation. The comparison of HIF1 ODD-luc and HRE-luc activators proves that all HIF ODD-luc stabilizers induce HRE-driven expression, but not *vice versa*. Comparison of the hits in HIF ODD-luc and Neh2-luc assay (Table 1A&B) clearly points to the distinct structural requirements for either reporter activation as presented below.

**Figure 2.**
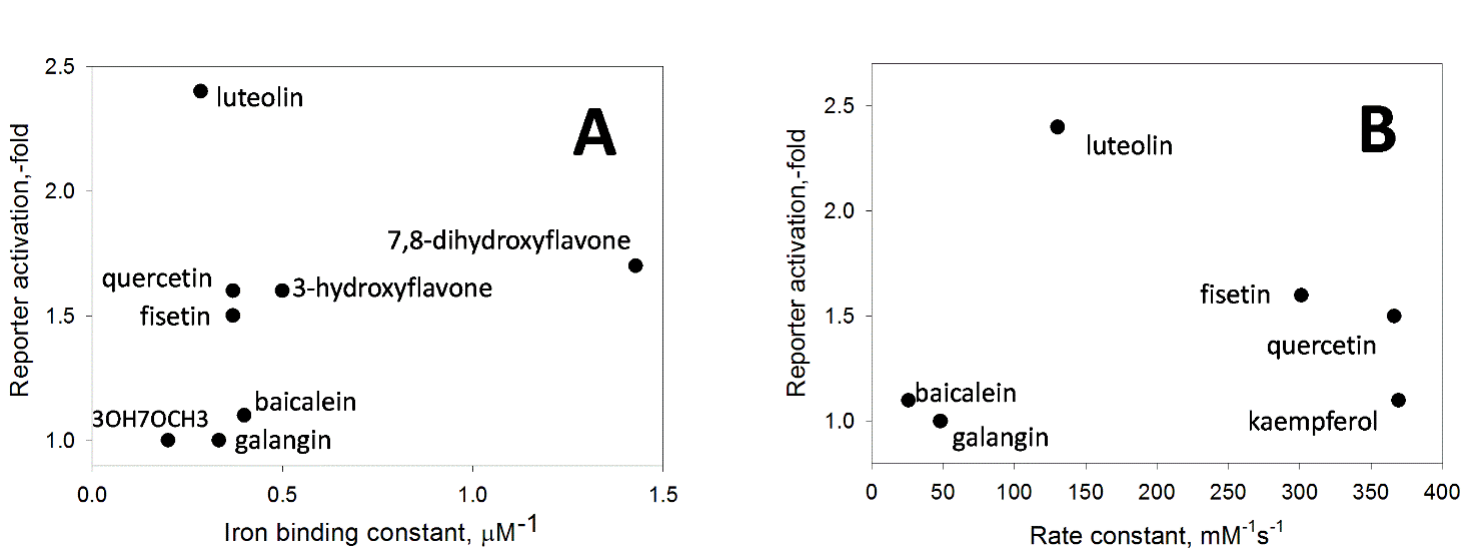
**Specificity for the flavone structure in HIF1 ODD-reporter activation**. **A**) Independence of activation amplitude from iron binding constant, and **B**) Independence of activation amplitude from ferricyanide (FC) reduction rate constant (The protocols for the determination of the constants under Materials and Methods).

### Structure-activity relationship (SAR) for activators of HIF1 ODD-luc reporter

#### Flavones

Distinguishing between iron non-specific chelation in solution and specific iron coordination inside the active center of HIF PHD is a challenge. To identify specific inhibitors we used the same approach we developed before for the comparison of branched oxyquinolines as HIF PHD inhibitors [26]. For compounds with iron binding properties, the judgment on the specificity for reporter activation (or enzyme inhibition *in vitro*) can be made from the plot of the reporter activation (or enzyme inhibition constant) versus the iron binding constant [26]. Non-specific inhibitors will show a linear plot with increasing activation numbers for increasing iron binding constants, whereas specific inhibitors will deviate (pop-up) from this tendency. As shown in Fig 2A, there is no linear dependence on flavones’ iron chelation ability for ODD HIF1-luc activation parameters, although all flavones shown in Fig 2A are iron chelators in the micromolar range. Luteolin and 3’,4’-dihydroxyflavone (#18 & 19, Table 1A) are clearly the best activators. 7,8-Dihydroxyflavone (#20) is the most potent iron chelator among all flavones tested, with iron dissociation constant of ca. 700 nM. 3-Hydroxyflavone (#1) has the constant of ca. 2 µM, while 5-hydroxyflavone does not bind iron in solution. The order of iron dissociation constant values is: CalceinAM << 7,8-Dihydroxyflavone << 3-hydroxyflavone < baicalein < quercetin=fisetin < 3’,4’-dihydroxyflavone < galangin = luteolin < 3-hydroxy-4’-methoxyflavone < 3-hydroxy-7-methoxyflavone (the latter has the dissociation constant of ca. 5 µM, #5 in Table 1A). The order of the activity increase in HIF1 ODD-luc reporter activation is: 7,8-Dihydroxyflavone<3-hydroxyflavone<calcein <fisetin<quercetin<luteolin=3’,4’-dihydroxy-flavone, with baicalein, galangin and 3-hydroxy-7-methoxyflavone (3OH7OCH3 in Fig. 2A) being inactive in the reporter assay. In HIF1 ODD-luc screen, calcein AM (#29) works no better than luteolin (#18), although their iron dissociation constants differ by 2 orders of magnitude (Table 1A and B), pointing again to the need in specific inhibition and not iron chelation in solution. Quercetin has been shown to directly inhibit HIF prolyl hydroxylase 2 [8]; its effect was ascribed by authors to its iron chelation ability in solution, based on comparison of inhibitory properties of 3-hydroxyflavone, 5-hydroxyflavone, 3’4’-dihydroxyflavone and their methoxy analogs, largely because of the inability of the existing *in vitro* capture assays to generate reliable quantitative data for iron chelators. Amgen’s capture assay protocol gives K_i_ of 10 µM for quercetin [7], which is a rather high estimate given the EC_50_ determined in HIF1 ODD-luc reporter assay (ca. 2.5 µM). In accord with the data obtained, luteolin is the best enzyme inhibitor among the flavones tested.

**Figure 3.**
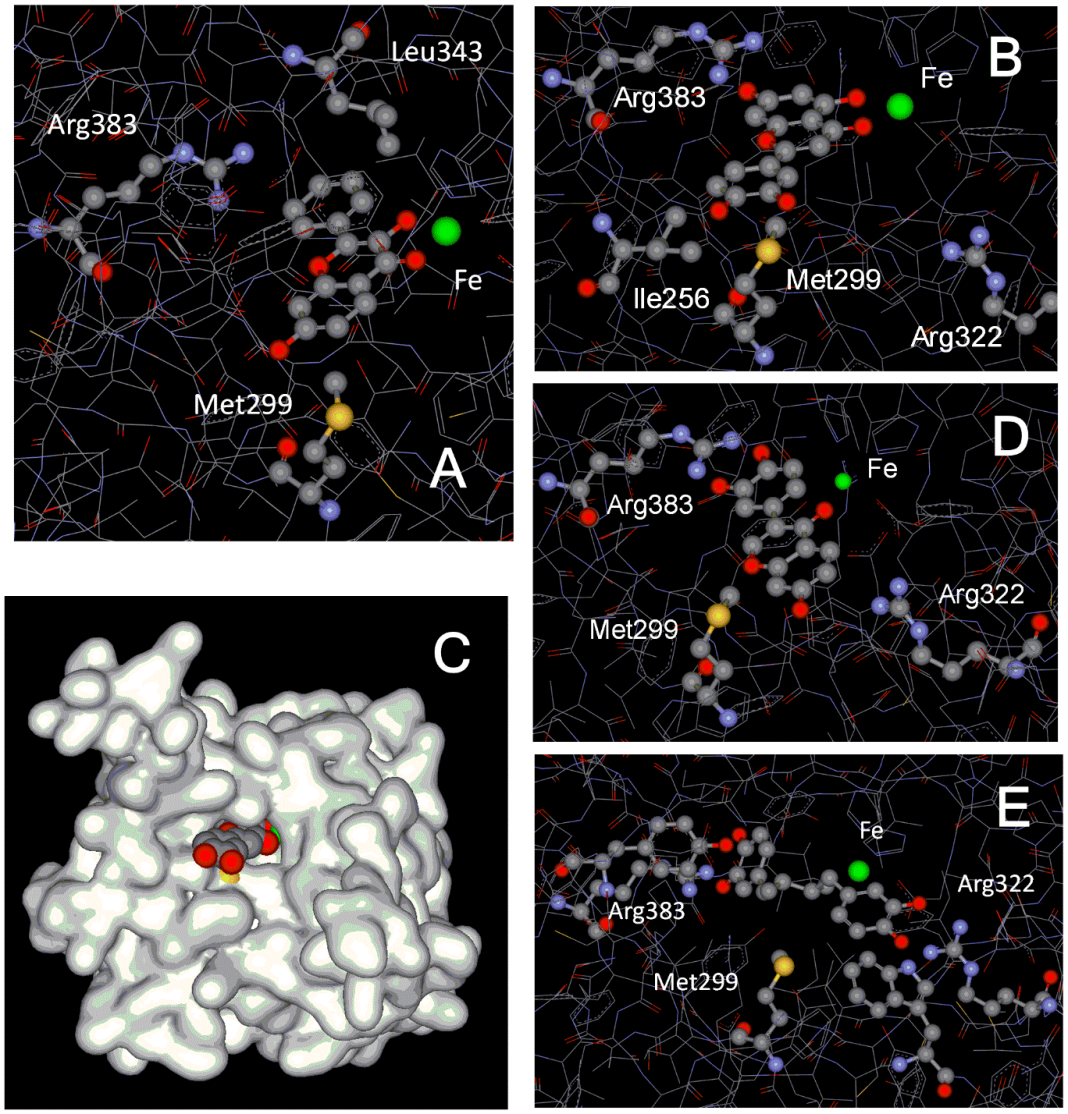
**Structural requirements for activating HIF1 ODD-luc reporter correspond to those for inhibiting HIF PHD2**. **A**) 3,7-hydroxyflavone docking into PHD2 active site illustrating bi-ligand iron chelation via carbonyl oxygen and 3-hydroxygroup and interaction of 5-hydroxygrop with Met299; **B**) docking of luteolin, active center view, and **C**) overall view; **D**) docking of 3’-hydroxydaidzein, and **E**) NDGA.

In addition to metal chelation properties, majority of flavones are potent reducing agents. One may expect that the intermediate oxo-iron form of HIF PHD can be reduced by a flavonoid, and hence, the enzyme is inhibited and HIF1 stabilized (HIF1 ODD-luc protein in the case of this reporter). To evaluate the reducing power of flavonoids, we have measured the rate constants for flavonoids oxidation with ferricyanide. If non-specific reduction of the enzyme intermediate had been the case, we would observe a linear dependence of reporter activation versus the rate constant for non-specific ferricyanide reduction by flavones. However, this dependence shows no linear trend (Fig. 2B), and again, luteolin pops-up among all other flavones tested. Therefore, neither iron chelation nor reducing properties of flavones determine HIF1 ODD-luc reporter activation. In other words, there are specific structural requirements for the reporter activation by flavones that are in agreement with docking predictions (Fig. 3) for HIF PHD2 inhibition, as follows. (a) 3-Hydroxyflavones are good activators as long as there are no substitutions in phenyl ring at positions 2’-4’ (compare compounds #1,2 vs. #3,4 and kaempferol #9 Table 1A); (b) 3-Hydroxygroup is critical for activation providing the iron chelation motif (compare #1 vs #6 & #7) (Fig. 3A); the presence of 5-hydroxygroup in addition to 3-hydroxygroup destabilizes iron coordination and decreases the activation effect (see #7 and galangin #8); (c) Substitutions in the phenyl ring for 3-hydroxyflavones give active compounds only in combination with 3’,4’-dihydroxy motif (fisetin #10, quercetin #11, myricetin #13) but not 2’,4’-dihydroxygroups (morin #12); (d) 5-Hydroxyflavone has no iron chelation properties in solution, and its derivatives are inactive independent of the presence of additional hydroxyl-groups in positions 6 and 7 (#14-17 Table 1A); (e) Only the presence of 3’4’-dihydroxy-group in phenyl ring providing interaction within the binding pocket of PHD2 gives an active 5-hydroxyflavone compound (luteolin, #18 in Table 1A, Fig. 3B,C). Of note, 3’4’-dihydroxy-flavone is as potent as luteolin (#19 Table 1A). Summarizing our findings, we conclude that catechol moiety in flavonoids is a must for inhibiting HIF PHDs in the absence of 3-hydroxy-substitution.

#### Isoflavones

In the case of isoflavones the structural requirements for HIF1 ODD-luc activation are less clear since the hit rate for isoflavones was much lower than for flavones. Daidzein (#26 Table 1B) did not cause any activation effect at short incubation times, whereas 3’-hydroxydaidzein (#27) was almost as potent as quercetin or luteolin (Table 1A), and this observation was supported by docking (Fig. 3D). 3’-Hydroxylation of flavonoids is known to be catalyzed by liver [31]. 3’-Hydroxydaidzein is one of the major metabolites of daidzein (in addition to equol). Comparison of 3’-hydroxydaidzein with 2 other trihydroxyisoflavones (##24,25) known for their higher iron binding and antioxidant activity (radical scavenging activity of 8’-hydroxydaidzein equals to that of alpha-tocopherol [32-34]) shows that the latter compounds are inactive in the low micromolar range (< 10 µM) (Table 1B). This observation again points to the fact that just radical scavenging activity is not sufficient to drive the antihypoxic program.

The effects generated by flavones/isoflavones in HIF1 ODD-luc screen could not be explained by their estrogenic activity: first, because this screen is specific for HIF PHD inhibitors, and second, the known ranking for the activation of estrogen receptors ERα (genistein > daidzein > apigenin > biochanin A = kaempferol > ipriflavone = quercetin = chrysin) and ERβ (genistein > daidzein > biochanin A = apigenin = kaempferol > quercetin = ipriflavone = chrysin) [5] does not correlate with their ranking in our systems. On the contrary, we believe that estrogens can work as HIF prolyl hydroxylase inhibitors because estradiol and estriol came up as poor hits in Spectrum library screen using HIF ODD-luc reporter system [26].

#### Catechols and NDGA

Dihydroxybenzoate (DHB) bearing a classic catechol motif, is a known inhibitor of HIF prolyl hydroxylase mimicking the α-KG binding mode in the enzyme active center: the DHB’s catechol motif provides two ligands for the HIF prolyl hydroxylase iron and DHB’s carboxy-group bids to Arg-383 deep inside the enzyme active center. DHB has the enzyme inhibition constant of 5 µM determined in the enzyme *in vitro* assay [35], but is a rather weak reporter activator working above 50 µM (Table 1B). The offset in the activation parameters originates from DHB competition with the intracellular α-KG (1 to 2 mM). A more than an order of magnitude offset of EC50 for cell-based reporter activation versus the value of the inhibition constant measured in the enzyme *in vitro* assay has been reported for all enzyme inhibitors mimicking α-ketoglutarate mode of binding [36]. Spectrum library had a number of levodopa analogs, and to our surprise, only levodopa by itself, but not *D*-DOPA or carbidopa, behaved as a rather potent HIF1 ODD-luc reporter activator with EC50=15 µM. (Table 1B). These results are supported by docking studies (not shown). Didox, the ribonucleotide reductase inhibitor currently in clinical trials for the cancer treatment, has two distinct iron binding motifs, and possibly, due to iron coordination by the hydroxamic acid motif, is a much weaker enzyme inhibitor working in the reporter assay only above 100 µM (Table 1B). Despite the fact that NDGA activates the reporter only 2-fold (Table 1B), it has an EC50 value of ca. 2 µM. In the docking studies, one catechol motif of NDGA interacts with the active site Arg-383, whereas the other interacts with Arg-322 at the active site entrance (Fig. 3E).

### LOX inhibition and VEGF induction

**Figure 4.**
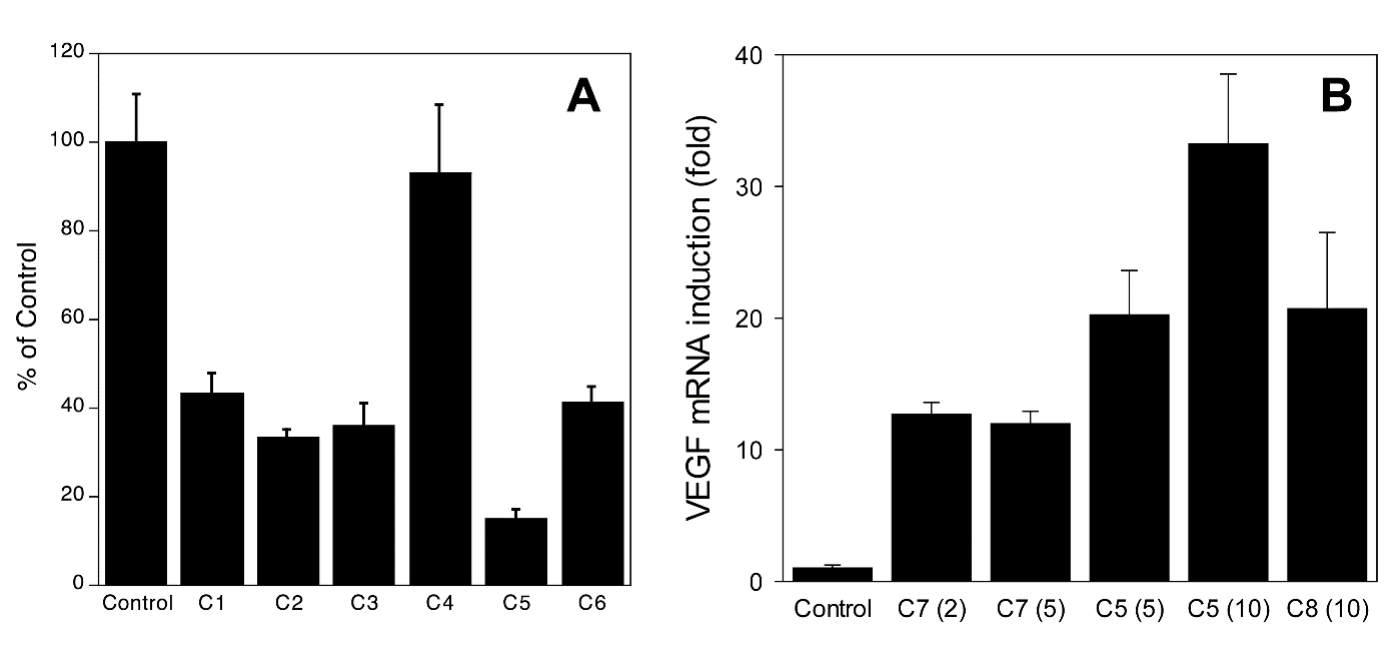
**Comparison of NDGA with flavonoids**. **A**) Inhibition of rabbit reticulocyte 15-LOX-1 activity; **B**) Induction of VEGF mRNA. C1, fisetin, C2, luteolin, C3, kaempferol, C4, genistein, C5, NDGA, C6, quercetin, C7, 3’4’-dihydroxyflavone, C8, FG-4592, used as a control HIF PHD inhibitor. For LOX inhibition compounds were used at 25 µM; for VEGF induction pretreatment was performed at concentrations shown in brackets in µM.

Comparison of flavonoids and NDGA in LOX inhibition assay demonstrates that all flavonoids except genistein are good LOX inhibitors, but less potent than NDGA (Fig. 4A). In addition to being a key redox enzyme in oxidative stress-related cell death, 12/15-LOX has also been shown to regulate HIF1 and induce VEGF [37, 38]. Comparison of one of the best flavones, 3’,4’-dihydroxyflavone to NDGA and FG-4592, a HIF PHD inhibitor developed by Fibrogen (Fig. 4B) demonstrates that flavones are mild inducers compared to FG-4592 and NDGA, and that the latter is at least 2-times more potent than FG-4592. This result may reflect the double nature of NDGA inhibitory effect targeting both LOX and HIF PHD.

### Neuroprotection in glutathione depletion model is in agreement with compounds’ ranking in HIF ODD-luc reporter assay

The prior studies on HIF PHD inhibition in a glutathione depletion model of oxidative stress in cortical neurons (homocysteinic acid model, HCA model) revealed that inhibition of HIF PHD1 by gene silencing or with canonical PHD inhibitors such DFO (iron chelator), DHB, and DMOG (α-KG mimics), was sufficient to prevent cell death independent of HIF1 [39]. The flavone hits identified in HTS with HIF1 ODD-luc reporter are definitely not PHD-isoform specific, therefore, one may expect that they will target PHD1 as well and exert neuroprotection in the above model in accord with their ranking in the reporter assay.

The structural requirements for HIF PHD2 inhibition are best exemplified by comparison of fisetin (“memory booster” [40] and luteolin (#10 & #18, respectively, Table 1A). Both compounds are potent reducing agents, and do not significantly differ in their ability to bind iron in solution (both have catechol moiety which is capable of binding iron). The combination of 3’4’- dihydroxy-groups with 3-hydroxyflavone (fisetin) interferes with the optimal docking into PHD2 and gives a lower reporter signal than their combination with 5-hydroxyflavone (luteolin) (Table 1A).

**Figure 5.**
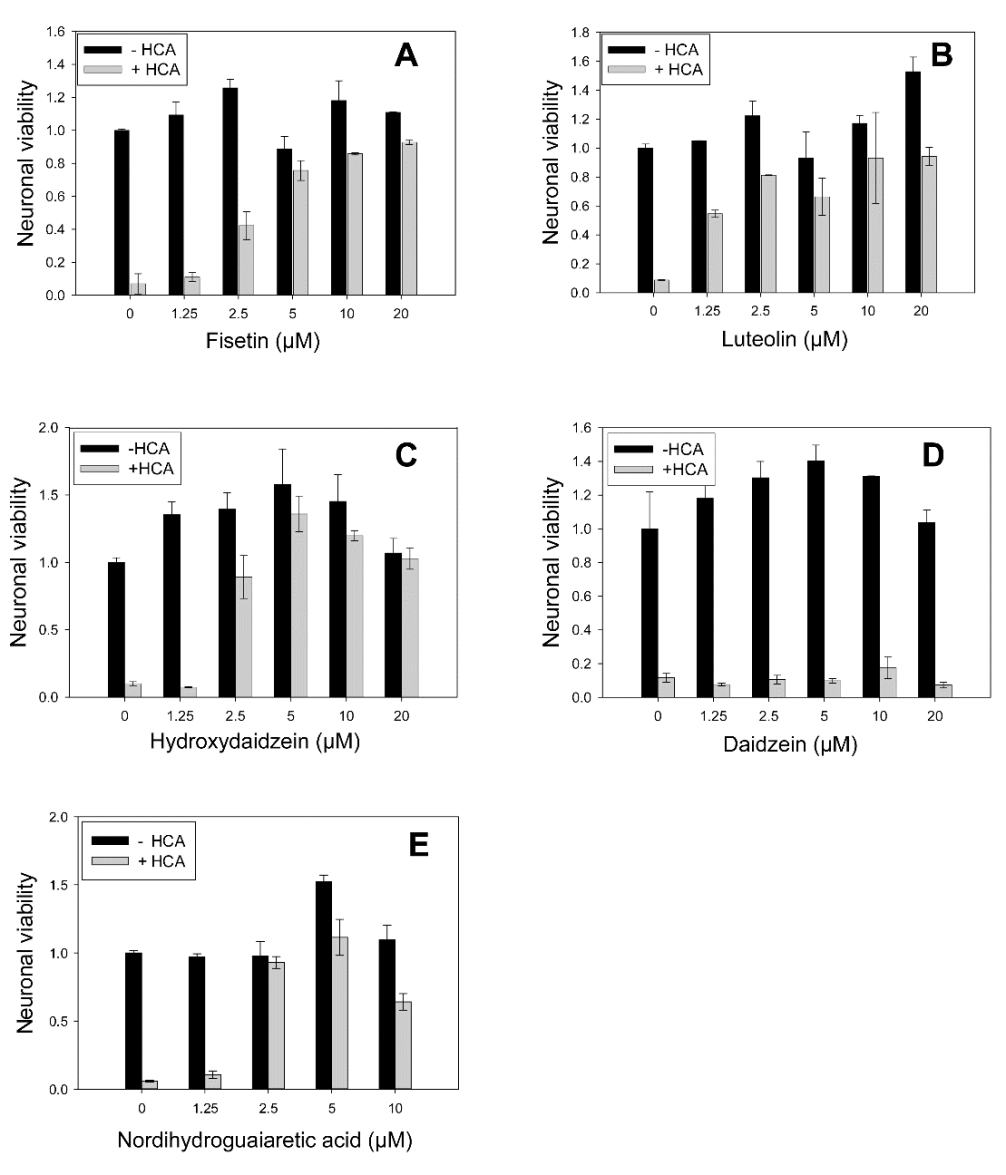
**Comparison of effects of flavonoids and catechols in glutathione depletion model**. Neuroprotective effects in HCA model correspond to compounds ranking in HIF ODD-luc screen (**A**, fisetin, **B**, luteolin, **C**, 3’-hydroxydaidzein, **D**, daidzein, **E**, NDGA).

Compound ranking in the glutathione depletion assay corresponds to that in HIF ODD-luc reporter activation: luteolin exerts neuroprotection in the glutathione depletion model already at 1.2 µM whereas fisetin begins to work at 2.5 µM (Fig. 5) despite the fact they are equipotent as LOX inhibitors. In the case of 3’-hydroxydaidzein and daidzein, the former is protective in the glutathione depletion model while the latter is not, consistent with the predictions based on the compounds’ activity in the reporter assay. Similarly, in accord with the ranking in the reporter activation assay, levodopa is protective in the glutathione depletion model with EC50= 6 µM, whereas carbidopa is non-protective up to 20 µM (results not shown). 3’-hydroxydaidzein and NDGA exhibit similar properties in HIF ODD-luc reporter activation (Table 1B) and in neuroprotection in the glutathione depletion model already at 2.5 µM (Fig. 5), despite the fact that NDGA is a more potent LOX inhibitor than any flavonoid. One may speculate that HIF PHD works upstream of LOX or belongs to a different, or parallel pathway having a major impact on cell survival under glutathione depletion conditions.

### Structure-activity relationship (SAR) for activators of Neh2-luc reporter

#### Flavones

**Figure 6.**
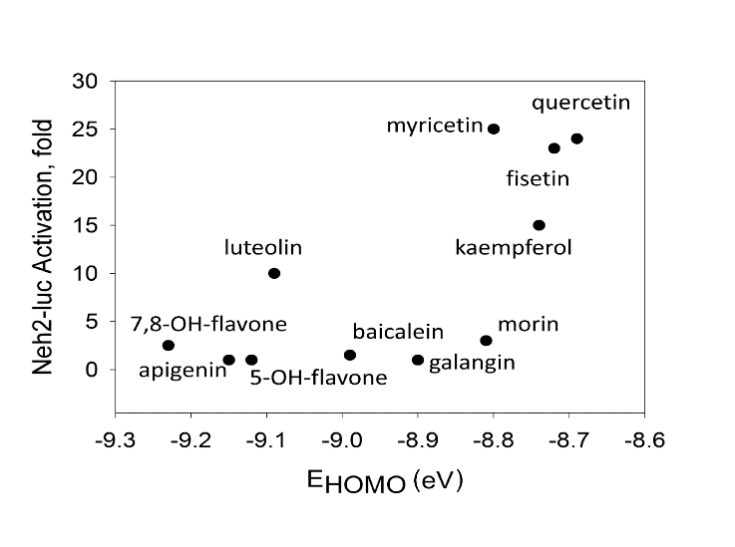
**Independence of Neh2-luc reporter activation from redox potential (E_HOMO_) of flavones**.

As could be seen from Table 1A, Neh2-luc reporter activation requires a combination of 3-hydroxygroup (forming a metal chelation motif) with a catechol type 3’,4’-dihydroxygroup, a pro-oxidant motif. There is no linear dependence on redox potential [41] of flavones (Fig. 6) pointing to the fact that Nrf2-Keap1 interaction is disrupted by specific flavones bearing the pro-oxidant motif in combination with zinc chelation motif. The activity exhibited by kaempferol may be considered as an argument against such interpretation, however, the simultaneous addition of N-acetyl cysteine (NAC), a potent cell-permeable reducing agent, with flavones has no effect on reporter activation by fisetin or quercetin, but exerts a 2-fold drop in reporter activation by kaempferol (results not shown). Quenching by NAC points to an additional oxidative modification by 3-hydroxylase taking place in the case of kaempferol. This enzymatic hydroxylation has specific steric requirements since morin bearing hydroxygroups in *para*- and *meta*-position (4’ and 2’) and thus having a restricted access to the 3’-position for catalytic hydroxylation is completely inactive in the reporter assay despite the fact that it has the same redox potential as fisetin, quercetin, and kaempferol (Table 1A). The latter three flavonoids have been previously reported as potent Nrf2 stabilizers and HO-1 inducers among other flavonoids tested (see Fig. 3, p.175 in [42]).

#### Isoflavones and catechols

As we demonstrated earlier, catechol by itself is an Neh2-luc reporter activator, which requires an additional modification, since there is a lag-period on the time course of reporter activation that cannot be shortened with increasing concentrations of the activator [27]. Didox is apparently a very weak Nrf2 activator, although it has the combination of catechol and zinc-binding motif (Table 1B). This may result from lower cell membrane permeability for negatively charged compounds. The same is true for levodopa and its analogs. In Neh2-luc reporter assay only isoflavones bearing a catechol motif were active: 3’-hydroxydaidzein was active, and daidzein not, again pointing to the pro-oxidant action of a catechol motif. 3’-Hydroxydaidzein properties in both reporter assays (Table 1B) strongly resemble NDGA, except NDGA is a much more potent and direct activator of Nrf2 similar to fisetin [27]. Like isoflavones, NDGA has estrogenic receptors as additional targets, and is the best inhibitor of LOX among the compounds studied in this work (Fig. 4A).

Nrf2 activators have been proved to benefit outcomes in Parkinson’s disease (PD) animal models, and given the fact that NDGA is much more stable *in vivo* than flavonoids undergoing cycle opening reactions, can reach decent concentrations in blood and crosses the blood-brain barrier, NDGA was chosen to run *in vivo* experiments. The additional advantage for choosing NDGA is that it also inhibits LOX and stabilizes HIF1 in the low concentration range. HIF1 has been recently shown to induce ATP13A2 (PARK9) [43], mutations of which cause an autosomal recessive form of early-onset parkinsonism (Kufor-Rakeb Syndrome) [44].

**Table 2:** Striatal levels of Dopamine and its metabolites

Treatment groups	N	DOPAMINE	DOPAC	HVA
CONTROL	5	89.9 ± 6.1	8.5 ± 0.76	7.8 ± 1.2
NDGA	5	88.4 ± 6.2	7.59 ± 1.35	7.5 ± 1.66
MPTP	10	35.4 ± 3.45*	3.92 ± 0.35*	4.7 ± 0.63*
NDGA+MPTP	10	55.3 ± 5.7#	6.04 ± 1.14#	7.4 ± 0.96#

The levels were measured by HPLC Electrochemistry. N is the number of animals in the group. Data in the table represent Mean ± SD. Values are ng/mg protein.

* *p* < 0.05 compared to control and

# *p* < 0.05 compared to MPTP.

### Neuroprotective effects of NDGA in the acute MPTP mouse model of Parkinson’s disease

**Figure 7.**
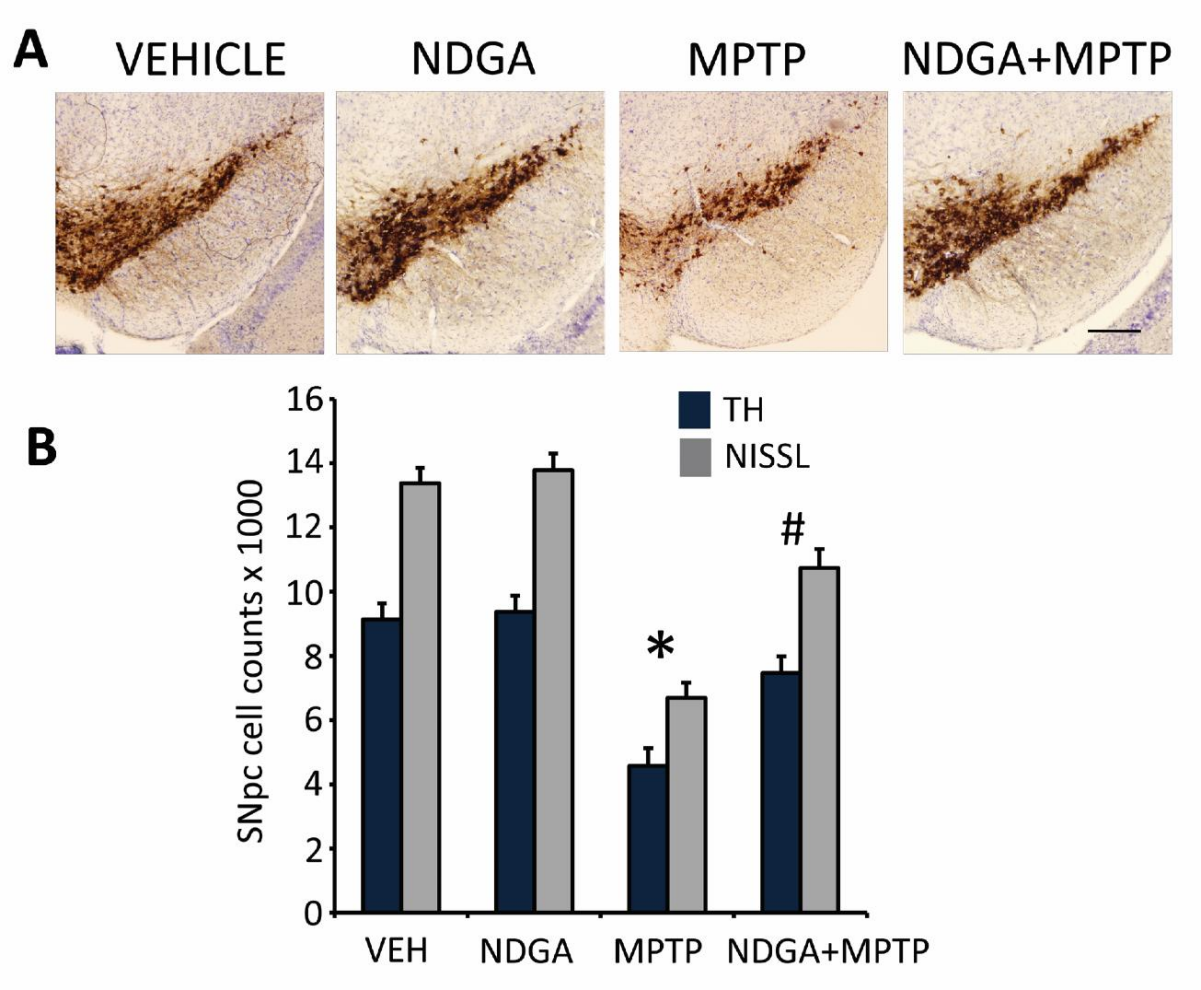
**Neuroprotective effects of NDGA in the MPTP model of Parkinson's disease**. (**A**) Immunohistochemical staining for TH and (**B**) stereological analysis of total (NISSL) and TH+-neurons in the SNpc in the acute MPTP model on the 7th day after treatment with NDGA. Bars represent mean ± SEM. *p < 0.05 compared to Vehicle controls, and #*p* < 0.05 compared to MPTP (*n*=8 mice per group).

Several lines of studies suggest that activation of Nrf2 mediated gene transcription either via a genetic or pharmacologic approaches have profound neuroprotective effects against MPTP-neurotoxicity [30, 45, 46]. Hence, we sought to test if activation of Nrf2/ARE signaling by NDGA can induce a neuroprotective response against MPTP-neurotoxicity. We used an acute paradigm of MPTP administration (10 mg MPTP/kg X 3, every two hours), known to cause about a 50% loss of striatal dopamine and its metabolites DOPAC and HVA and significant loss of tyrosine hydroxylase (TH) immunopositive neurons in the substantia nigra pars compacta (SNpc) on the 7^th^ day. Evaluation of neuroprotective effects of NDGA against MPTP neurotoxicity found that pretreatment of NDGA at 100mg/kg/day but not 50mg/kg/day protected against MPTP-induced loss of striatal dopamine, DOPAC and HVA (Table 2). Consistent with levels of striatal catecholamines unbiased stereologic counts of total (i.e. Nissl-positive) and TH-positive neurons in SNpc showed a statistically significant loss of neurons in the MPTP group compared to controls (Fig. 7). Analysis of total and TH-positive neuronal counts for NDGA, when administered at 100mg/kg/day, showed a significant attenuation of MPTP-induced loss of total (i.e. Nissl-positive) and TH-immunopositive neurons as compared to MPTP treated mice (Fig. 7). The doses that were used to determine the efficacy of NDGA against MPTP were determined by evaluation of brain HO-1 levels. Both 50 and 100 mg/kg administered twice a day induced a dose dependent increase in HO-1 mRNA levels at 6 hours after last dose of NDGA. At 100 mg/kg HO-1 levels showed a 6-fold increase whereas 50 mg/kg only induced a 1.2 fold increase in HO-1 mRNA levels (data not shown). The neuroprotective effects of NDGA against MPTP-neurotoxicity were not due to impaired metabolism of MPTP to its toxic metabolite MPP+ (1-methyl-4-phenyl-pyridinium ion) as judged by HPLC-fluorimetric analysis of striatal MPP+ levels measured 90 minutes following a single intraperitoneal injection of 30 mg/kg free base MPTP: no significant difference between the MPTP and NDGA+MPTP groups (Table 3). Collectively these results suggest a neuroprotective effect of NDGA in a mouse model of MPTP-induced PD.

**Table 3:** Striatal MPP+ levels

Treatment groups	MPP+
MPTP	8.1 ± 0.14
NDGA+MPTP	8.4 ± 0.22

The levels were measured following MPTP and NDGA+MPTP at 90 min. Data in the table represent Mean ± SD. Values are ng/mg weight of the striatal tissue (n=5 mice per group). The values are not significant between MPTP and NDGA + MPTP, therefore significance is not shown.

## DISCUSSION

There is a popular opinion that flavonoids are good iron chelators and potent antioxidants and this is sufficient to drive the antihypoxic and antioxidant response. This study demonstrates that there are distinct structural requirements for activation of either program at the step of transcription factor stabilization. The structural requirements for activation of HIF ODD-luc reporter correspond to those necessary to inhibit HIF prolyl hydroxylase, whereas structural requirements for stabilization of Nrf2 require the combination of 3-hydroxy- and 3’4’-dihydroxy- groups.

The ranking of luteolin and fisetin in Neh2-luc activation (fisetin, the best one) is completely opposite to that for HIF1 ODD-luc activation (luteolin, the best one) again pointing to the need in a chelation motif for Nrf2 activation. A logical speculation is that flavones target cysteine residues coordinating Zn^2+^ atom in Keap1 [47] resulting in significant conformational changes in the latter. The 3-hydroxy group is not obligatory for Neh2-luc activation, since 3’,4’-dihydroxyflavone is an activator with the potency comparable to kaempferol (Table 1A). However, just zinc coordination ability without pro-oxidant catechol motif is insufficient for the reporter activation since 3-hydroxyflavone is completely inactive (Table 1A).

It is interesting to note that ARE-luc screen used to rank flavonoids as Nrf2 activators came to the same conclusion on structural requirements, despite the authors claimed linear dependence of flavone activity on redox potential [41]. This discrepancy reflects imperfections of ARE-luc screen, with prolonged incubation times being the major disadvantage, which in the case of flavonoids results in monitoring the effect of their oxidative products as well. A good illustration to the above statement is soy isoflavone daidzein, which is supposed to be beneficial for survival under various stress conditions [48, 49]. However, based on our results, large portion of daidzein benefits one must ascribe to its major metabolite, 3’-hydroxydaidzein, the direct activator of both survival programs.

No doubt the dietary flavonoids are beneficial for intestines, where their bioavailability can reach dozens of µM, contrary to plasma concentrations reaching just 0.5 µM, which is not sufficient to drive the antihypoxic or antioxidant program, especially in the brain. Low bioavailbility of flavonoids drives medicinal chemistry efforts to design their structural analogs of higher bioavailability with uncompromised biological activity in specific *in vitro* models [50]. Despite the known low bioavailability and stability, quercetin in 25-75 mg/kg dose twice a day for 4 days was shown to be neuroprotective in the rotenone-induced hemiparkinsonian rats [51]. Our choice of NDGA to run *in vivo* animal model for PD, and not a flavonoid, was based on the understanding of these limitations of flavonoids bioavailability on one hand, and the “multi-target” advantage of NDGA similar to the best flavonoids from this study on the other hand. MPTP-model is a toxic model and we suppose that superior Nrf2 activator properties of NDGA in combination of potent inhibition of LOX play a major role in neuroprotection. As we predicted, NDGA was demonstrated to be neuroprotective in a PD animal model.

### Conclusion

All flavonoids are commonly considered as powerful cell survival agents, but the precise mechanisms for this remain obscure. Using two novel reporters we demonstrate that there are distinct structural requirements for direct stabilization of HIF and Nrf2 transcription factors by flavonoids. We show that 3’-hydroxydaidzein, but not daidzein itself, is a direct trigger of antihypoxic and antioxidant programs. We choose NDGA, a natural catechol with high stability and bioavailability, with similar properties to 3’-hydroxydaidzein, for *in vivo* studies in Parkinson’s disease model, and demonstrate its neuroprotective effects.
